# Childhood stunting and wasting in Myanmar: Key drivers and implications for policies and programmes

**DOI:** 10.1111/mcn.12710

**Published:** 2020-08-24

**Authors:** Jessica L. Blankenship, Jennifer Cashin, Tuan T. Nguyen, Hedy Ip

**Affiliations:** ^1^ UNICEF East Asia and Pacific Regional Office Bangkok Thailand; ^2^ Alive & Thrive Southeast Asia, FHI 360 Yangon Myanmar; ^3^ Alive & Thrive Southeast Asia, FHI 360 Hanoi Vietnam; ^4^ UNICEF Myanmar Yangon Myanmar

**Keywords:** Demographic and Health Survey (DHS), Myanmar, stunting, wasting, young children

## Abstract

Child undernutrition is a public health and development problem in Myanmar that is jeopardizing children's physical and cognitive development and the country's social and economic progress. We identified key drivers of child stunting (low height‐for‐age) and wasting (low weight‐for‐height) in a nationally representative sample (*n* = 3,981) of children 0–59 months of age. The national prevalence of child stunting and wasting was 28% and 7%, respectively. Boys were more likely to be stunted or wasted than girls. Older children 24–35 months were at the highest risk of stunting compared with children under 6 months (risk ratios [RR] 10.34; 95% CI [6.42, 16.65]) whereas the youngest, under 6 months, were at the highest risk of wasting compared with children 36–59 months (RR 2.04; 95% CI [1.16, 3.57]). Maternal height <145 cm (RR 5.10; 95% CI [3.15, 8.23]), perceived small child size at birth (RR 2.08; 95% CI [1.62, 2.69]), and not benefiting from institutional delivery (RR 1.52; 95% CI [1.24, 1.87]) were associated with an increased risk of child stunting, as were maternal occupation, unimproved household drinking water, living in delta, coastal or upland areas, and poorer household wealth index quintile. Increased risk of child wasting was associated with maternal underweight (RR 1.64; 95% CI [1.11, 2.42]) and open defecation (RR 1.91; 95% CI [1.25, 2.92]) as well as maternal occupation and residence in a coastal area. Our findings indicate that the key drivers of child undernutrition in Myanmar are multifaceted and start in utero. Investing in scaling‐up multisectoral approaches that include nutrition‐specific and nutrition‐sensitive interventions with a focus on improving maternal nutrition is essential for reducing child undernutrition and contributing to further gains in the country's human and economic development.

**Key messages**

- In Myanmar, the prevalence of stunting steadily increases during the 0‐ to 23‐month period. The effects of stunting during the first 1,000 days are largely irreversible.
- Early child growth restriction, poor maternal nutrition, and inadequate coverage of maternal health services are leading drivers of child undernutrition.
- Household and environmental drivers associated with child undernutrition include low household wealth index, lack of access to safe drinking water, and the practice open defecation.
- A multisectoral approach with a focus on maternal nutrition and the first 2 years of life is essential to improve children's nutritional status in Myanmar.

## 1 INTRODUCTION

Child undernutrition is a global development problem resulting from poor access to nutritious foods, repeated infections, and inadequate maternal and child feeding and care practices during the first 1,000 days, from conception to age two. Undernutrition has major implications for children's physical growth and cognitive development as well as for a country's human, social, and economic progress. While chronic undernutrition, as measured by the prevalence of stunting (low height‐for‐age), is gradually declining in the countries of the Southeast Asian (ASEAN) region, an estimated 18 million children under 5 years in the region have stunted growth and are at risk of failing to reach their growth and developmental potential (ASEAN/UNICEF/WHO, 2016). The prevalence of acute undernutrition, as measured by wasting (low weight‐for‐height), has largely been stagnant in the region, with at least 5.4 million children affected.

Since 2011, the Republic of the Union of Myanmar (hereafter referred to as Myanmar) has undergone substantial political, economic, and administrative reforms, with shifts to democratic governance and a market‐based economy (World Bank, 2014). The removal of economic sanctions and the increases in foreign investment and development assistance have, in part, led to improvements in living standards for much of the population. However, despite rapid economic growth, one in three individuals in Myanmar lives below the national poverty line of 1,241 Kyat per capita (US$ 1.21; World Bank, 2017), and child undernutrition continues to be a public health concern, with over 1.3 million children under five stunted and more than 300,000 wasted at any given time (DoP, 2015; Ministry of Health and Sports & ICF, 2017; UNICEF, 2017).

There is compelling evidence that addressing undernutrition during a child's first 1,000 days is one of the most cost‐effective development actions for poverty reduction and economic growth (Copenhagen Consensus Center, 2015). Undernutrition in utero and during early childhood increases both child mortality and morbidity (Prado & Dewey, 2012) and contributes to productivity losses through impaired cognition, reduced schooling attainment, and lower work productivity (Horton & Steckel, 2013). Childhood stunting contributes to an intergenerational cycle of undernutrition, a vicious cycle of deprivation that reduces the ability of populations to escape poverty (Hoddinott et al., 2011).

Although the predictors of child undernutrition are well‐documented globally (Bhutta et al., 2008), they have not been explored in Myanmar. Small‐scale subnational surveys suggest that poor maternal nutrition status, low birth weight, suboptimal infant and young child feeding practices, and frequent disease are important risk factors for child undernutrition in the country (Phyo, Keiwkarnka, & Mongkolchati, 2014; Save the Children, 2014). In this study, we investigated the drivers of child stunting and wasting in children under 5 years of age using nationally representative data from the Myanmar's 2015–2016 Demographic and Health Survey (DHS).

## 2 METHODS

### 2.1 Sampling methodology and data collection

The Myanmar Demographic and Health Survey (MDHS) report contains a detailed description of the survey design, sample selection, survey tools, and data collection methods (Ministry of Health and Sports & ICF, 2017). To summarize, the sample for the MDHS was designed to provide estimates for indicators on the situation of women and children in urban and rural areas for each of the country's 14 states and regions and for the Nay Pyi Taw Union Territory. The sample was based on the 2014 census frame, and the survey was conducted in 12,500 households (97.8% response rate).

Three questionnaires were utilized in the MDHS: a household questionnaire, a women's questionnaire, and a men's questionnaire. The household questionnaire collected basic information on household characteristics and anthropometry of children under 5 years and women of reproductive age (15–49 years). The women's questionnaire collected socio‐demographic characteristics of mothers and information on birth history, antenatal, delivery and post‐natal care, breastfeeding and infant and young child feeding practices, and childhood illnesses for children under 5 years of age.

### 2.2 Statistical methods

Descriptive statistics for the prevalence of stunting, wasting, and concurrent stunting and wasting were generated based on the WHO (2006) Child Growth Standards according to which a child with height‐for‐age z‐score (HAZ) < −2 is considered to be stunted, a child with a weight‐for‐height z‐score (WHZ) < −2 is considered to be wasted, and a child with both HAZ < −2 and WHZ < −2 is considered to be both stunted and wasted. Bivariate analyses were conducted to examine differences in the prevalence of stunting and wasting according to child, maternal, and household characteristics based on the UNICEF conceptual framework for the determinants of child undernutrition (UNICEF, 2013).

Child factors assessed included child sex, child age (as a dummy variable to allow for nonlinear associations), and perceived size at birth. Maternal factors assessed included age at delivery, years of education, occupation, receipt of antenatal care (ANC) services, institutional delivery, nutrition status measured by body mass index (BMI), and stature. Household environmental factors included wealth index, access to improved drinking water and sanitation, and area of residence.

Multinomial logistic regression models were created to compare the children who were stunted only, and wasted only with children who had normal nutrition status (no stunting, wasting, or underweight). We excluded children who were both stunted and wasted (*n* = 61) and overweight (*n* = 66) because of their small sample size. The models were adjusted for the variables of child age, child sex, maternal age, maternal education, perceived child size at birth, and other variables that had a significant association in bivariate analysis with child stunting or wasting. Children with a missing height or weight measurement and those with a HAZ or WHZ score <−6 or >6 (WHO, 2006) were excluded from the analysis. Sampling weights were utilized to account for the unequal probability of selection resulting from the sample design and nonresponse. Results are expressed as relative risk ratios (RR) with 95% confidence intervals. A *P* value of less than 0.05 was considered statistically significant. Statistical analyses were conducted using SPSS version 22.0 (Armonk, NY: IBM Corp).

## 3 RESULTS

There were 3,981 children 0–59 months of age included in the analysis with all child, maternal, household, and environmental variables (sample size and characteristics for all variables are shown in Table 1). The prevalence of stunting increased steadily with child age during the period from 0 to 35 months, with the highest prevalence rate found among children 24–35 months of age (Table 2). The overall prevalence of stunting was higher in boys (30.3%) compared with girls (26.3%; *P* < 0.01), with significant differences between the sexes in the 6‐ to 11‐month‐old (*P* < 0.01) and 12‐ to 23‐month‐old (*P* < 0.001) age groups. The overall prevalence of child wasting was 7.7% among boys and 6.4% among girls (Table 2). The prevalence of wasting was highest among infants 0–5 months of age regardless of gender, with the prevalence of wasting more than two times higher among girls (18.5%) compared with boys (7.0%) 0–5 months of age (*P* < 0.01) and no sex differences in wasting prevalence among older children. These results illustrate that a substantial proportion of children in Myanmar are undernourished during the critical 1,000‐day period, from conception to 2 years of age, the effects of which have been found to be largely irreversible.

**Table 1:** Selected characteristics of the study sample^a^

	Mean ± *SD* (%)		Mean ± *SD* (%)
Maternal characteristics (*n* = 3108)^a^		Child characteristics (*n* = 3,981)	
Age (years)	31.5 ± 6.7	Age (months)	29.7 ± 17.1
Education attainment (years)	5.7 ± 4.1	Male child	51.7
BMI (kg/m^2^)	22.8 ± 4.2	Institutional delivery of child	40.2
Height (m)	1.5 ± 0.1	Mother's perceived size of the child at birth	
<145	6.3	Do not remember	3.9
145–149	24.0	Smaller than normal	12.0
150–159	61.9	Normal	60.1
≥160	7.8	Larger than normal	24.0
Nutrition status		Household characteristics (*n* = 3108)	
Underweight (BMI < 18.5)	12.1	Wealth index quintile^b^	
Normal weight (18.5 ≤ BMI < 25)	60.6	Poorest	26.9
Overweight (BMI ≥ 25)	21.3	Poorer	22.5
Obese (BMI ≥ 30)	6.0	Middle	18.3
Occupation		Richer	17.5
Skilled manual labourer	5.2	Richest	14.9
Farm labourer	29.2	Area (% urban)	22.4
Self‐employed	9.1	Use of improved water	80.4
Professional, sales and services	20.9	Open defecation	13.1
Not employed	35.6	Agroecological zone^c^	
		Uplands	18.4
		Delta	38.3
		Coastal	9.5
		Dry	33.8

*Note*. BMI: body mass index.

a The sample included 3,981 children under 5 years old, born by 3,108 mothers who lived in 3,108 households. Only mothers of sampled children were included in analysis (other caregivers excluded).

b Wealth index quintiles were developed using principal components analysis summarizing housing variables (e.g., roof, walls, and floor) and asset variables (e.g., television, radio, and car).

c Agroecological zone—Uplands: Chin, Kayin, Kayah, Kachin, and Shan; Delta: Ayeyarwaddy, Yangon, Bago, and Mon; Coastal: Rakhine and Tanintharyi; Dry: Magway, Mandalay, Naypyitaw, and Sagaing.

**Table 2:** Prevalence (%) of stunting and wasting by age and sex

Age category (months)	*n*	Normal^a^			Stunted^b^			Wasted^c^		
		Male (%)	Female (%)	Total (%)	Male (%)	Female (%)	Total (%)	Male (%)	Female (%)	Total (%)
0–5	408	85.0	75.6*	80.6	7.6	5.8	6.8	7.0	18.5**	12.6
6–11	410	72.3	87.3**	79.6	20.1	7.5**	14.1	7.6	4.6	6.3
12–23	808	59.5	76.9***	67.4	30.2	16.2***	23.8	10.5	6.9	8.8
24–35	780	52.1	54.4	53.3	41.2	41.4	41.3	6.7	4.1	5.3
36–47	847	59.5	62.0	60.7	34.4	34.4	34.4	6.2	3.5	4.9
48–59	728	60.2	61.6	60.9	32.4	31.8	32.1	7.5	6.6	7.0
Total	3981	62.0	67.2**	64.5	30.3	26.3**	28.4	7.6	6.4	7.1

*Note*. Statistical testing for differences between male and female children. NS: not significant.

a Normal defined as the child did not suffer from underweight, stunting, or wasting.

b Stunted defined as height‐for‐age z‐score (HAZ) < −2.

c Wasted defined as weight‐for‐height z‐score (WHZ) < −2.

* *P* < 0.05.

** *P* < 0.01.

*** *P* < 0.001 (chi‐square, adjusted for sampling design).

### 3.1 Drivers of child stunting and wasting

Drivers of increased risk of child stunting included child age, child sex, perceived child size at birth, maternal stature, maternal occupation, institutional delivery, mother's occupation as a farm labourer or working in an office or shop, institutional delivery, household wealth index, household drinking water, and area of residence (Table 3). Children perceived by mothers as being smaller than normal at birth were more likely to be stunted (RR 2.08; 95% CI [1.62, 2.69]), compared with those perceived to be normal size at birth. Women with height <145 cm were five times more likely to have a stunted child (RR 5.10; 95% CI [3.15, 8.23]); however, all women with a height of <160 cm had increased risk of having a stunted child. Populations in delta, coastal and upland zones, in the poorest three wealth quintiles, and those consuming drinking water from an unimproved source were also more likely to have a stunted child in the household (*P* < 0.05).

**Table 3:** Associations between child, maternal and household factors, and stunting and wasting in children 0–59 months of age (*n* = 3,981)^a^

	Stunted^b^ RRR [95% CI]	Wasted^c^ RRR [95% CI]
Child characteristics		
Age (months)		
0–5 (reference)	1	1
6–11	2.34 [1.36, 4.01]**	0.49 [0.28, 0.86]*
12–23	4.29 [2.65, 6.95]***	0.81 [0.52, 1.25]
24–35	10.34 [6.42, 16.65]***	0.61 [0.37, 1.01]
36–47	7.43 [4.63, 11.93]***	0.49 [0.30, 0.81]*
48–59	6.70 [4.14, 10.84]***	0.65 [0.39, 1.07]
Male child	1.37 [1.16, 1.62]***	1.50 [1.13, 1.99]**
Mother's perceived size of the child at birth		
Do not remember	1.45 [0.96, 2.20]	2.49 [1.40, 4.40]**
Smaller than normal	2.08 [1.62, 2.69]***	1.31 [0.86, 2.00]
Larger than normal	0.78 [0.63, 0.96]*	0.54 [0.36, 0.80]**
Normal (reference)	1	1
Maternal characteristics^d^		
Age (years)	1.00 [0.99, 1.02]	1.02 [0.99, 1.04]
Maternal education (years attained)	0.99 [0.97, 1.02]	1.00 [0.96, 1.05]
Maternal height (cm)		
<145	5.10 [3.15, 8.23]***	0.67 [0.30, 1.51]
145–149	2.83 [1.87, 4.29]***	1.00 [0.58, 1.74]
150–159	1.96 [1.32, 2.91]**	0.87 [0.53, 1.44]
≥160 (reference)	1	1
Nutrition status		
Normal (18.5 ≤ BMI < 25; reference)	1	1
Underweight (BMI < 18.5)	1.23 [0.95, 1.58]	1.64 [1.11, 2.42]*
Overweight (BMI ≥ 25)	0.81 [0.65, 1.02]	0.78 [0.52, 1.16]
Obese (BMI ≥ 30)	0.58 [0.38, 0.88]*	1.00 [0.54, 1.84]
Occupation		
Skilled manual labourer	1.47 [0.97, 2.24]	0.38 [0.16, 0.95]*
Farm labourer	1.35 [1.09, 1.67]*	0.64 [0.44, 0.94]*
Self‐employed	1.20 [0.89, 1.63]	0.89 [0.52, 1.53]
Professional, sales, and services	1.49 [1.16, 1.90]*	0.83 [0.56, 1.22]
Not employed (reference)	1	1
No institutional delivery of child	1.52 [1.24, 1.87]***	1.15 [0.81, 1.62]
Household characteristics
Wealth index quintile		
Poorest	1.79 [1.21, 2.65]**	0.92 [0.49, 1.71]
Poorer	1.55 [1.07, 2.26]*	0.87 [0.49, 1.54]
Middle	1.51 [1.04, 2.20]*	1.13 [0.66, 1.95]
Richer	1.21 [0.85, 1.74]	0.71 [0.42, 1.21]
Richest (reference)	1	1
Urban area	0.87 [0.67, 1.13]	1.48 [0.98, 2.24]
Unimproved drinking water	1.27 [1.02, 1.56]*	1.15 [0.80, 1.66]
Practice of open defecation	0.76 [0.58, 0.99]*	1.91 [1.25, 2.92]**
Agroecological zone		
Uplands	1.38 [1.09, 1.75]**	0.89 [0.57, 1.38]
Delta	1.27 [1.03, 1.57]*	1.12 [0.78, 1.59]
Coastal	1.39 [1.02, 1.91]*	1.65 [1.03, 2.64]*
Dry (reference)	1	1

*Note*. Data were presented as relative risk ratio (RRR) and 95% confidence interval (95% CI). Statistically differ from null value (RRR = 1) with the following:

* *P* < 0.05.

** *P* < 0.01.

*** *P* < 0.001.

a Multinomial logistic regression with baseline comparison group as children with normal nutrition status (no stunting, wasting, or underweight). All estimations are adjusted for survey design and sampling.

b Stunted defined as height‐for‐age z‐score (HAZ) < −2.

c Wasting defined as weight‐for‐height z‐score (WHZ) < −2.

d Only mothers of sampled children were included in analysis (other caregivers excluded).

Drivers for increased risk of child wasting included child sex, perceived child size at birth, maternal underweight, practice of open defecation, and residence in a coastal zone (Table 3). Children perceived by mothers to be larger than normal at birth had a lower risk of wasting (RR 0.54; 95% CI [0.36, 0.80]), compared with children perceived to be normal size at birth. Mothers who were underweight were more likely to have a wasted child (RR 1.64; 95% CI [1.11, 2.42]). Households practicing open defecation were more likely to have a child affected by wasting (RR 1.91; 95% CI [1.25, 2.92]). Finally, children whose mothers worked as skilled manual labourers or as farm labourers had a significantly lower risk of wasting compared with children of mothers who were not employed.

## 4 DISCUSSION

Despite rapid social and economic growth in recent years, child undernutrition remains a public health and development problem in Myanmar and represents a major barrier to the country's future development. Results of this analysis indicate that the age of the child modified the risks of child stunting and wasting and that periods of highest risk coincide with critical stages of children's physical and cognitive development.

The risk of stunting was highest among children 24–35 months of age, which likely reflects the cumulative effect of growth faltering in utero and in early childhood (WHO, 2014). Children who remain stunted after 2 years of age have missed the window of opportunity during the first 1,000 days from conception to 2 years of age, in which the effects of poor growth and development are reversible. Children stunted after 2 years of age are at increased risk for poor cognitive, educational, and productivity outcomes throughout life (Fink & Rockers, 2014). Although individual catch‐up growth later in childhood and adolescence is possible (Crookston et al., 2013), this has not been documented at the population level (Leroy, Ruel, Havicht, & Frongillo, 2015).

The much higher prevalence of wasting among children under 6 months of age is consistent with findings from other ASEAN countries, where a high burden of wasting in the youngest infants has been largely attributed to poor breastfeeding practices, low maternal BMI, and low weight gain during pregnancy (Institute for Public Health, 2008; NSO, 2016). The implications of wasting in children under 6 months are serious, and potentially irreversible, even if children later recover. Wasting not only increases the risks of infant morbidity and mortality (Olofin et al., 2013), but there is a strong evidence correlating the occurrence of wasting during the first 6 months of life to cognitive delays and or permanent cognitive damage (Grantham‐McGregor, 1995; Hoffman, Sawaya, Verreschi, Tucker, & Roberts, 2000; Martins et al., 2011). Additionally, wasting during the critical period of 12–23 months of age, as shown in our study, is a known risk factor for child stunting, with repeated episodes of wasting during this time contributing to growth faltering (Richard et al., 2012).

Though the prevalence of wasting was higher among girls than among boys in the 0‐ to 5‐month age group, boys had a higher overall prevalence of both wasting and stunting. These findings are consistent with a meta‐analysis of data from 84 countries, which showed that boys were more likely to be stunted, wasted, and concurrently stunted and wasted than girls (Khara, Mwangome, Ngari, & Dolan, 2017). The sex disparity in undernutrition has largely been attributed to the greater biological vulnerability of boys, who also experience increased mortality and morbidity compared with girls (Wamani, Astrom, Peterson, Tumwine, & Tylleskar, 2007); however, further analysis is required to better understand inequity of undernutrition by child sex.

Our study revealed that poor maternal nutrition status was a key driver of child stunting and wasting, illustrating the intergenerational impact of undernutrition in Myanmar. This finding supports those found in South Asian countries (Vir, 2016) where the most important risk factors influencing child stunting relate primarily to maternal characteristics. Maternal undernutrition, characterized by low prepregnancy BMI, shorter maternal height, and inadequate gestational weight gain, is an important contributor to intrauterine growth restriction (IUGR) in under‐resourced countries with IUGR being the leading global risk factor for stunting in children (Danaei et al., 2016). An estimated 32% of child stunting and 30% of child wasting may be attributed to IUGR, as measured by small for gestational age (Danaei et al, 2016; Black et al., 2013). In our study, more than one in five children were reportedly smaller than normal size at birth, a proxy indicator for IUGR, and these children had a higher risk of stunting. In addition, shorter maternal height, indicative of stunting during the mother's childhood, was a driver of child stunting, with all maternal stature <160 cm associated with a higher risk of stunting. This is consistent with findings from a meta‐analysis of data from 109 country DHS surveys, which identified an inverse association between maternal height and the prevalence of child stunting (Ozaltin, Hill, & Subramanian, 2010).

Coverage of essential maternal health services, particularly ANC and institutional delivery, is limited in Myanmar, with only 40% of women in our study delivering in a health facility. Children born at home were more likely to be stunted, with similar findings reported in India (Biswas & Bose, 2011), Nepal (Tiwari, Ausman, & Agho, 2014), and Bangladesh (Rahman & Chowdhury, 2007), where women who had an institutional delivery were more likely to receive information on exclusive breastfeeding and complementary feeding and essential newborn care. Our analysis further substantiates that poor coverage of health services is associated with a higher prevalence of child stunting (Kuhnt & Vollmer, 2017). Access to adequate health care in line with the WHO recommendations on ANC for a positive pregnancy experience (WHO, 2016), along with improving women's nutrition, can have a transformative impact on children's height. Provision of these essential maternal and child health services during the first 1,000 days has the potential to interrupt the vicious cycle of malnutrition within a single generation (Garza et al., 2013).

In Myanmar, as in other low‐ and middle‐income countries, there are substantial inequalities between regions and population subgroups. We found that lower household wealth index was significantly associated with higher odds of child stunting, with children in the poorest households nearly two times more likely to be stunted compared with children in the wealthiest households. Consistent with findings from other DHS surveys, we did not find an association between the prevalence of wasting and household wealth quintile (Assaf & Pullum, 2016). Previous research has suggested that immediate factors, such as child feeding and care practices and prevalence of disease and inflammation, are drivers of child wasting and are mediating factors between socio‐economic status and child wasting (Fernandez, Himes, & de Onis, 2002; Zayeri et al., 2016). Agroecological zone was significantly associated with child stunting in Myanmar, with children in the mountainous upland states most likely to be stunted and children in the coastal zone more likely to be wasted, a finding that is in line with previous national and subnational surveys (LIFT, 2016; Ministry of National Planning and Economic Development & Ministry of Health, 2011). The upland states are characterized by remoteness and poor accessibility, vulnerability to food insecurity (WFP, 2012; Solidarites International, 2012; LIFT, 2013), lower educational attainment (Department of Population, 2015), and higher rates of poverty (World Bank, 2014). The coastal zone is characterized by poor sanitation, unimproved drinking water (WHO, 2015), high prevalence of helminth infection (Tun, Myat, Gabrielli, & Montresor, 2013), and frequent exposure to disasters caused by natural hazards (LIFT, 2013).

Environmental factors such as unimproved water and sanitation are the second largest global drivers of child stunting (Danaei et al. 2016). Our finding that the consumption of unimproved household drinking water was associated with a higher risk of child stunting and the practice of open defecation was associated with a greater risk of child wasting is consistent with previous findings. Contaminated drinking water and inadequate sanitation are important contributors to infection and diarrheal disease. Over time, chronic exposure to faecal pathogens, without manifesting as diarrhoea, may lead to subclinical environmental enteropathy with potentially deleterious effects on nutrient absorption and use and children's linear growth (Humphrey, 2009).

Our study has some limitations. In our sample, recorded birth weight was present on the immunization card in 45% cases only. Therefore, we used of mother's perceived size of the child at birth as a proxy indicator for birth weight and presence of IUGR (Channon, 2011). Use of mother's perceived size of the child at birth has the potential for recall bias and social desirability bias for this variable. Other proxy indicators of IUGR, such as maternal weight gain during pregnancy, were not collected in the survey.

Poverty is an important risk factor for child stunting (Danaei et al. 2016). Yet our findings indicate that poverty reduction, alone, will not address the fully child undernutrition in Myanmar. The main drivers of child undernutrition in our analysis suggest the need to prioritize investments during the first 1,000 days, with a focus on improving maternal nutrition, increasing coverage of maternal health services, improving access to clean water and safe sanitation, and creating an enabling environment that includes legislation, policies, and programmes to support improved behaviours and practices for good nutrition and sanitation.

Global evidence suggests that integrating a set of evidence‐based interventions into existing platforms and programmes, such as ANC, maximizes the impact on children's nutritional status and results in significant reductions in the prevalence of child undernutrition (Bhutta et al., 2013). Improving both the coverage and quality of ANC, with a particular focus on counselling early and consistently during pregnancy on key nutrition actions such as weight gain during pregnancy, dietary diversity, and micronutrient supplementation for the mother, as well as appropriate infant and young child feeding practices, child care, and stimulation, will have a positive impact on maternal and child nutrition status (Kim et al., 2016; Menon et al., 2016).

The association between child undernutrition and indicators of poverty, including lack of access to safe drinking water and sanitation, underscores the need for multisectoral approaches and investments in nutrition‐sensitive strategies, with clearly defined nutrition‐specific goals, in the areas of agriculture, water and sanitation, rural development, social protection, health, and education, to achieve accelerated and sustained reductions in child undernutrition (Ruel, 2013). Implemented at scale, the combination of nutrition‐specific interventions and multisectoral approaches have been shown to have a dramatic impact on stunting reduction (Remans, 2011). Other multisectoral interventions, such as maternity protection and workplace policies, further enable mothers to achieve optimal breastfeeding (Rollins et al., 2016), child feeding, and child care practices and are important to ensure that social and economic development in Myanmar translates into improved nutrition outcomes for mothers and children.

In conclusion, a combination of interventions delivered at critical points during the first 1,000 days is needed to address the critical drivers of child undernutrition in Myanmar and ensure that the country's economic development translates into improved health and nutrition outcomes for mothers and children. Scaling‐up both nutrition‐specific and nutrition‐sensitive interventions with a focus on improving maternal nutrition status before and during pregnancy will improve child nutrition, thereby contributing to human capital development and economic gains for the country.

## CONFLICTS OF INTEREST

The authors declare that they have no conflicts of interest.

## CONTRIBUTIONS

The authors' responsibilities were as follows: JLB, JC, TTN, and HI designed the study; JLB and TTN acquired and analysed the data; JLB, JC, TTN, and HI interpreted the data; JLB and JC drafted this manuscript; and JB, JC, TTN, and HI provided critical intellectual feedback to help revise the manuscript. All authors have read and approved the final manuscript.
